# Two cases of extensively drug-resistant (XDR) *Neisseria gonorrhoeae* infection combining ceftriaxone-resistance and high-level azithromycin resistance, France, November 2022 and May 2023

**DOI:** 10.2807/1560-7917.ES.2023.28.37.2300456

**Published:** 2023-09-14

**Authors:** Clara Maubaret, François Caméléna, Manel Mrimèche, Aymeric Braille, Mathilde Liberge, Mary Mainardis, Clémence Guillaume, Franck Noel, Cécile Bébéar, Jean-Michel Molina, Florence Lot, Emilie Chazelle, Béatrice Berçot

**Affiliations:** 1 Paris Cité University, INSERM1137, IAME, Paris, France; 2 APHP, Infectious Agents Department, Saint Louis - Lariboisière University Hospitals, Paris, France; 3 French National Reference Centre for bacterial STI, Associated Laboratory for Gonococci, Paris, France; 4 Orléans Hospital, Microbiology Laboratory, Orléans, France; 5 Bioxa Laboratoire, Microbiology Laboratory, Bezannes, France; 6 University of Bordeaux, USC EA 3671 Mycoplasmal and Chlamydial Infections in Humans, Bordeaux, France; 7 University Hospital, Bacteriology Department, French National Reference Centre for Bacterial STIs, Bordeaux France; 8 Paris Cité University, INSERM, UMR S976, Paris, France; 9 AP-HP, Infectious Disease Department, Saint Louis – Lariboisière Hospitals, Paris, France; 10 Santé publique France, the national public health agency, Saint-Maurice, France; * These authors contributed equally to this work and share first authorship.

**Keywords:** XDR, gonorrhea, penA-60.001, A2059G mutation, France, sexually transmitted infections, bacterial infections, gonorrhoea, multidrug resistance, laboratory surveillance, laboratory, molecular methods, typing

## Abstract

We report two extensively drug-resistant (XDR) *Neisseria gonorrhoeae* (NG) isolates combining high-level resistance to azithromycin and resistance to ceftriaxone, obtained in France from two heterosexual patients, one of whom returned from Cambodia. Whole genome sequencing identified MLST ST16406, the mosaic *penA*-60.001 which caused ceftriaxone resistance in the internationally spreading FC428 clone, and the A2059G mutation in the 23S rRNA gene. The NG isolates F93 and F94 were related to XDR isolates detected in Austria and the United Kingdom in 2022.

The emergence of extensively drug-resistant (XDR) *Neisseria gonorrhoeae* (NG) is of major global public health concern. Infections with XDR NG can result in treatment failure and limited options for antibiotic treatment [1,2]. In 2022, two NG isolates with high-level azithromycin resistance and ceftriaxone resistance were identified in Europe, one in Austria and the other in the United Kingdom (UK) [3,4].

We describe here the, to the best of our knowledge, first two XDR NG strains isolated in France, in November 2022 and May 2023, combining high-level resistance to azithromycin and resistance to ceftriaxone, cefixime, ciprofloxacin and tetracycline.

## Clinical case descriptions

### Case 1

The first case occurred in November 2022 in a heterosexual woman consulting the emergency department of a general hospital in France for an upper genital tract infection. She reported frequent unprotected sex with the same partner, no travel within the 6 months before consultation and no history of prior sexually transmitted infections (STIs). The woman’s partner was not available for examination and no personal information could be obtained from the partner despite our requests.

A vaginal swab was performed for microbiological research, and antibiotic treatment with ceftriaxone, doxycycline and metronidazole was initiated immediately, in accordance with French recommendations for upper genital tract infection [5]. Despite this procedure, the patient continued to experience pain and a fever, with laboratory signs of infection (C-reactive protein: 61 mg/L (normal value: > 5 mg/L); leukocytes 11 g/L (normal value: 4–11 g/L). The patient was transferred to the surgery department 1 day after admission, for exploratory laparoscopy. The surgeons removed the appendix and noted the presence of a purulent liquid in the rectouterine pouch and pseudomembranes in contact with the right fallopian tube and the patient was treated with ceftriaxone at a dose of 1 g daily for 3 days. Pharyngeal and anal swabs were not taken before treatment.

Samples were sent to the laboratory and analysed for opportunistic bacteria and *N. gonorrhoeae* using cultures [5]. Nucleic acid amplification testing (NAAT) was used to detect the presence of *N. gonorrhoeae* and *Chlamydia trachomatis* (Allplex STI essential assay, Seegene). By culture, the NG was detected on the intrauterine device 2 days after the operation only but not in the intraoperative liquid or on the vaginal swab. Antimicrobial susceptibility testing of the NG isolate showed it to be XDR including ceftriaxone resistance (Table). By NAAT, NG was detected in the vaginal swab and in the intraoperative liquid 2 days after the operation. Tests for *C. trachomatis* were negative. Two weeks after the operation, the patient underwent tests of cure at three sites (anal, vaginal and pharyngeal), with all three tests yielding negative results. 

**Table:** Minimum inhibitory concentrations of 12 antimicrobial drugs for two high-level azithromycin-resistant and ceftriaxone-resistant extensively drug-resistant *Neisseria gonorrhoeae* F93 and F94 isolates, France, November 2022 and May 2023

Antimicrobial drug	F93 (Case 1)		F94 (Case 2)	
	MIC in mg/L	Interpretation (EUCAST v 12.0)	MIC in mg/L	Interpretation (EUCAST v 12.0)
Azithromycin	> 256	Resistant (high-level)	> 256	Resistant (high-level)
Ciprofloxacin	8	Resistant	4	Resistant
Tetracycline	32	Resistant (high-level)	32	Resistant (high-level)
Doxycycline	16	Resistant	16	Resistant
Cefixime	1	Resistant	1	Resistant
Ceftriaxone	0.25	Resistant	0.25	Resistant
Cefotaxime	1	Resistant	1	Resistant
Spectinomycin	16	Susceptible	8	Susceptible
Gentamicin	4	NA (wild-type MIC)	4	NA (wild-type MIC)
Ertapenem	0.016	NA (wild-type MIC)	0.016	NA (wild-type MIC)
Rifampicin	0.064	NA (wild-type MIC)	0.125	NA (wild-type MIC)

EUCAST: European Committee on Antimicrobial Susceptibility Testing; MIC: minimum inhibitory concentration; NA: not applicable (because of lack of breakpoints for interpretation).

Interpretation according to EUCAST v 13.0, 2023 [8]. Wild-type MIC is the MIC usually observed for *N. gonorrhoeae* not resistant to the antibiotic tested.

### Case 2

The second case occurred in May 2023 in a heterosexual man living with HIV (undetectable viral load) consulting his general practitioner (GP) for urethritis. He described first symptoms of urethritis following unprotected sexual intercourse with a woman in Cambodia. No diagnosis was made at this time, and he was treated with 200 mg doxycycline daily for 7 days and a single dose of 1 g azithromycin while in Cambodia. He had not had any symptoms for 3 to 4 days. A few days later (at the beginning of May), on his return to France, the patient consulted for a relapse of symptoms. He declared that he had not had sex since his return to France. A urethral swab yielded positive results for NG on culture and NAAT, but the NAAT was negative for *C. trachomatis* (Allplex STI essential assay, Seegene). Antimicrobial susceptibility testing of the isolate obtained showed it to be XDR NG (Table). Pharyngeal and anal swabs was not taken before treatment. The patient received 1 g of ceftriaxone monotherapy as a single intramuscular dose in the middle of May as recommended by the French national guidelines [6], dosing which has been shown to be effective even on isolates with an MIC to ceftriaxone between 0.125 and 0.5 mg/L [7]. His symptoms subsequently resolved, and tests of cure performed 1 month later were negative for all three sites tested (genital, pharyngeal and anal). The patient did not have sexual intercourse during this period.

## Microbiological investigation

The NG isolates, F93 (Case 1) and F94 (Case 2), were cultured from an intrauterine device and a urethral swab, respectively, on PolyViteX agar (bioMérieux, France) in a humid atmosphere containing 5% CO_2_ for 24 h at +36 °C. They were sent to the Associated Laboratory of the French National Reference Centre for Bacterial STIs in Paris for expertise. The identification of the isolates was confirmed by MALDI-TOF mass spectrometry (Vitek MS, bioMérieux). Minimum inhibitory concentrations (MICs) for 11 antimicrobial agents were determined by Etests (bioMérieux) interpreted in accordance with European Committee on Antimicrobial Susceptibility Testing (EUCAST) recommendations [8]. The isolates were screened for the presence of a beta-lactamase with a nitrocefin disk test (Mast Group Ltd., UK).

Both isolates displayed high-level resistance to azithromycin (MIC > 256 mg/L) and resistance to cefixime, ceftriaxone, ciprofloxacin and tetracycline (Table). Neither isolate produced a β-lactamase. According to international definitions, the strains were classified as XDR.

## Molecular investigation

Genomic DNA was extracted with a QIAsymphony (QIAGEN) and the DSP DNA Mini Kit. Whole-genome sequencing and bioinformatic analysis were performed as previously described [9], and the sequencing reads obtained are available from the European Nucleotide Archive (ENA)/GenBank (Accession number: PRJNA998219). We aligned the genomes of F93 and F94, ceftriaxone-resistant strains previously isolated in France (WHOY (F89), F90, F91 and F92) and Japan (WHOX) and strains with high-level resistance to azithromycin and resistance to ceftriaxone recently described in the UK (WHOQ and H22–494 (Case 10)), Austria (AT158) and Australia (A2735 and A2543) [3,4,9-14]. We corrected the alignments for recombination with Parsnp software version 1.2 [15] and constructed a maximum-likelihood phylogeny for core-genome comparisons, visualised with iTol, as previously described [9].

The isolates F93 and F94 were assigned to multilocus sequence typing (MLST) sequence type (ST) 16406. They were assigned to different new STs by NG multi-antigen sequence typing (NG-MAST) (*porB* 822, *tbpB* 294 and *porB* 952, *tbpB* 294) and by NG sequence typing for antimicrobial resistance (NG-STAR) to ST4465 (NG-STAR profile: 60.001_89_13_1_1_176_1) and to a new ST (NG-STAR profile: 60.001_89_100_1_7_3_1). Both strains had acquired the mosaic allele *penA*-60.001 encoding a penicillin-binding protein 2 (PBP2) with amino acid substitutions (i.e A311V, N513Y) decreasing the rate of extended-spectrum cephalosporin (ESC) acylation of PBP2 and thus resulting in resistance to these antibiotics. The two isolates also harboured the A39T mutation of the MtrR protein, without promoter deletions, resulting in overexpression of the MtrCDE efflux pump and an increase in the MIC of ESCs [16]. In addition, F93 and F94 contained the A2059G mutation in the four alleles of the 23S rRNA-encoding gene (*rrl*) conferring the high-level of resistance to azithromycin [17]. The quinolone resistance-determining regions carried S91F and D95G (for F93) or D95A (for F94) substitutions in GyrA (subcomponent of DNA gyrase) and a S87R substitution in ParC (a component of topoisomerase IV), accounting for the high level of resistance to ciprofloxacin. The *tetM* genes were detected, and the *rpsJ* genes contained a mutation conferring the V57M amino acid substitution in the S10 ribosomal protein, contributing to tetracycline resistance.

The F93 isolate had 1,394 single nucleotide polymorphisms (SNPs) relative to F94. However, F93 was found to be closely related to the recently reported high-level azithromycin-resistant and ceftriaxone-resistant XDR isolates AT159 from Austria and H22–494 from the UK (Case 10), from which it differed by only nine and two SNPs, respectively. On the phylogenetic tree, F93 and F94 belonged to the same lineage as these previously identified XDR isolates. However, this lineage contained multiple sublineages: the A2543 XDR clone previously reported to be spreading internationally [4,11,18], the sublineage including AT159, H22–494 and F93, and a new sublineage corresponding to F94 (Figure).

**Figure:**
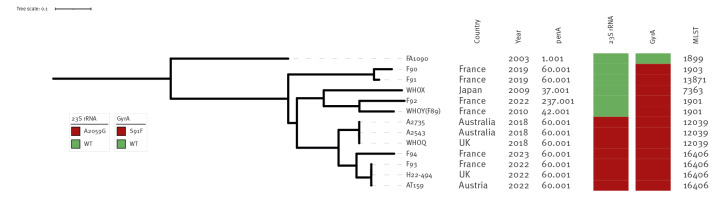
Ceftriaxone-resistant and high-level azithromycin-resistant *Neisseria gonorrhoeae* isolates, including the XDR-F93 and -F94 isolates from France, November 2022 and May 2023

MLST: multilocus sequence typing; UK: United Kingdom.

Core genome maximum likelihood phylogeny based on mapping onto the reference genome of the FA1090 strain with correction for recombination. On the basis of 10,225 single nucleotide polymorphisms, the high-level azithromycin-resistant and ceftriaxone-resistant *N. gonorrhoeae* F93 and F94 isolates are compared with previously described ceftriaxone-resistant and extensively drug-resistant *N. gonorrhoeae* isolates from Europe, Australia and Japan [3,4,12,13,19]. The tree is rooted on the FA1090 strain.

## Discussion

Five ceftriaxone-resistant and high-level azithromycin-resistant XDR NG isolates have been described worldwide to date: three in 2018 (A2543 and A2735 (Australia) and G7944/97687 (UK)) and two in 2022 in Europe (AT159 (Austria) and H22–494 (UK)) [3,4,12,13,19]. Here, we report two further isolates from France (the third and fourth isolates to be described in the European region since 2022), confirming the recent emergence of several XDR NG isolates in Europe. They combine ceftriaxone resistance from the mosaic *penA*-60-associated FC428 clone with the 23S rRNA A2059G target mutation conferring high-level resistance to azithromycin and resistance to ciprofloxacin and tetracycline. The emergence of these XDR strains with high-level resistance to azithromycin and resistance to ceftriaxone is a serious concern [18]. Four of the XDR isolates (including F93 and F94) recently described in Europe belong to MLST ST16406, with F94 separated from F93, AT159 and H22–494 by 1,394 SNPs. In contrast, the three XDR isolates A2543 and A2735 (isolated in Austria in 2018) and WHOQ (a UK case linked to travel to Thailand in 2018) belonged to MLST ST12039, and they differ only by one SNP [3,4,12,13,19]. However, the genomes of these seven XDR isolates were closely related, corresponding to the same lineage related to the A2543 clone initially described [3,4,12,13,19].

Five of the seven cases of infection with high-level azithromycin-resistant ceftriaxone-resistant strains were linked to travel in South-East Asia, indicating that they may be an epidemiological link to this area. One recent study highlighted the extensive dissemination in China of NG with the *penA*-60.001 allele conferring decreased susceptibility to cephalosporins, necessitating strict surveillance [20]. These findings suggest that these strains may continue to circulate in Asia while escaping detection. A recent report from the Enhanced Gonococcal Surveillance Programme (EGASP) highlights an urgent need for ongoing and expanded enhanced culture-based antimicrobial resistance surveillance to enhance the understanding of antimicrobial resistance evolution and spread in Cambodia [21]. Tests-of-cure, for the pharyngeal site in particular, culture and antimicrobial susceptibility testing are required to ensure successful treatment and surveillance.

## Conclusion

The spread of high-level azithromycin-resistant and ceftriaxone-resistant XDR NG clones may compromise the recommendations for the first-line treatment of gonococcal infections currently in place, highlighting a critical need for new effective therapies and international collaborations to survey antibiotic resistance. The newly developed antimicrobial drugs gepotidacin, lefamulin and zoliflodacin may be useful as alternative treatments for infections with XDR NG strains. The development of a vaccine against NG would provide an additional option for disease control in the future. In the meantime, it is essential to strengthen preventive actions to promote the use of condoms and screening in case of condomless intercourse or symptoms. People travelling to Asia should receive specific prevention information by travel medicine specialists, STI clinics or GPs.
